# Association Between Lower Levels of Vitamin D and Inflammation in the Geriatric Population: A Systematic Review and Meta-Analysis

**DOI:** 10.7759/cureus.60892

**Published:** 2024-05-23

**Authors:** Saud Salman Alharbi, Abdulaziz A Albalawi, Abdullah M Al Madshush, Waseem Mutlaq H Alsaidalani, Ohud S Aljohani, Abdulmohsen R Alaradi, Abrar A Alatawi, Rawabi S Albalawi, Lama A Alanazi, Hadeel S Albalawi, Ahmad E Asiri, Mohammed S Zamel, Saud Hussain

**Affiliations:** 1 Department of Family and Community Medicine, University of Tabuk, Tabuk, SAU; 2 Faculty of Medicine, University of Tabuk, Tabuk, SAU; 3 Faculty of Medicine and Surgery, University of Tabuk, Tabuk, SAU; 4 Faculty of General Medicine and Surgery, University of Tabuk, Tabuk, SAU; 5 Department of Internal Medicine, King Khalid University, Abha, SAU; 6 Department of Family Medicine, King Khalid University, Abha, SAU; 7 College of Medicine, King Khalid University, Abha, SAU

**Keywords:** geriatrics, meta-analysis, elderly, inflammation, vitamin d

## Abstract

There have been suggestions that vitamin D has anti-inflammatory effects; however, the variabilities of vitamin D levels among specific groups of patients and its association with these inflammatory events have not been demonstrated. This study aims to study the association between vitamin D levels and vitamin D deficiency and inflammatory events among the elderly population. PubMed, Web of Science, Scopus, Science Direct, and ClinicalKey were systematically searched in December 2023 to include the relevant data. Comprehensive Meta-Analysis (version 3.0, Biostat, Inc., Englewood, NJ) was the software used for data analyses. A total of 12 studies were included in this analysis with 14,717 elderly patients. There was an overall significant decrease in vitamin D levels in elderly patients with high inflammatory markers compared to controls (Hedges' g = -0.221, 95% CI: -0.268, -0.173, P < 0.001), and event of vitamin D deficiency was found to be 0.321 (95% CI: 0.305, 0.337, P < 0.001). There is a significant decrease in vitamin D levels among the elderly with different inflammatory conditions. Future longitudinal studies and well-designed, large, randomized controlled trials are required to study the association between vitamin D deficiency and the incidence of inflammatory events in this specific group of patients.

## Introduction and background

The conventional understanding of vitamin D is that it controls calcium and phosphorus to promote normal bone mineralization. Interest in vitamin D's involvement in extraskeletal processes, including inflammation and immunoregulation, has grown in light of recent findings [1,2]. Vitamin D plays an increasingly important role in inflammation [1].

Inflammation is a natural response by the body to injury or infection, but chronic inflammation can have detrimental effects on health, particularly in older adults. Research has found that low levels of vitamin D are linked to increased levels of inflammatory markers in the blood, such as C-reactive protein (CRP) and interleukin-6 (IL-6). These markers are associated with conditions like cardiovascular disease, diabetes, and arthritis, which are more prevalent in the geriatric population [2]. Vitamin D deficiency has been linked to the pathophysiology of a number of inflammatory diseases, encompassing rheumatoid arthritis and Crohn's disease, as well as conditions like obesity, insulin resistance, type 2 diabetes, and cardiovascular disease that are associated with low-grade chronic inflammation [3].

The aging process is linked to several alterations in the body; within this framework, low-grade systemic inflammation has been thoroughly investigated [4]. Recent studies on the elderly have demonstrated the impact that systemic inflammation plays in several outcomes, including comorbidity, frailty, impairments, and, eventually, death anticipation. Although various theories have been proposed, there is still disagreement regarding the elements that contribute to the onset of this inflammatory process [5].

Given that vitamin D insufficiency is still common around the globe and is rising as a result of sedentary indoor lives, the use of sunscreen, and protective clothing to lower the risk of skin cancer, the relationship between vitamin D and inflammation may have clinical ramifications [6]. Although there is now disagreement among specialists over the ideal amounts of vitamin D, most agree that plasma 25-hydroxyvitamin D (25(OH)D) levels less than 50 nmol/L would be deemed inadequate [7]. The fact that 10-40% of Americans and 20-60% of Britons have vitamin D levels below 50 nmol/L raises concerns, as stated in a previous study [8]. In Australia, vitamin D deficiency is common in 50% of women and 31% of men despite the country's sunny climate [9].

A previous systematic review demonstrated that the anti-inflammatory properties of vitamin D were repeatedly demonstrated in human cell lines and peripheral blood mononuclear cells. Cellular investigations are necessary to look at how 25(OH)D affects inflammatory states and responses [10]. Another study found that the majority of the studies that were examined were prospective and methodologically structured to evaluate the performance of 25(OH)D during an acute inflammatory response. In addition, following an inflammatory insult, the majority of them showed a swift drop in serum 25(OH)D levels [11]. A systematic review and meta-analysis provided level 1 evidence of the positive impact of vitamin D supplementation on inflammatory markers in type 2 diabetes. To determine if changes in inflammation after vitamin D administration might lead to clinically meaningful health outcomes for these patients, larger and longer-term clinical trials are required [12].

Maintaining adequate levels of vitamin D through supplementation or sun exposure may help reduce inflammation and lower the risk of chronic diseases in older adults. Further research is needed to fully understand the mechanisms underlying the association between vitamin D and inflammation in the geriatric population. This systematic review and meta-analysis examine the association between vitamin D levels and vitamin D deficiency and inflammatory events among the elderly population.

## Review

Methodology

Literature Search

The Preferred Reporting Items for Systematic Reviews and Meta-Analyses (PRISMA) criteria are followed in this meta-analysis [13]. A thorough systematic search of the literature was conducted in PubMed, Web of Science, Scopus, Science Direct, and ClinicalKey in December 2023. Our search was restricted to the English language and customized as needed for each database. The following keywords, which were converted into MeSH terms in PubMed or subject terms in Scopus, were used to identify the relevant study articles: “Vitamin D,” “25-hydroxyvitamin D,” “25 (OH) D,” “Deficiency,” “Inflammation,” “Inflammatory markers,” “Cytokines,” “Interleukins,” “C-reactive protein,” “Elderly,” and “Geriatric population.” Boolean operators like "OR" and "AND" were paired with the relevant keywords.

Study Selection and Data Extraction

The output of the search technique was double-checked using Rayyan (QCRI, Cambridge, MA) [14]. By modifying the combined search results with inclusion/exclusion criteria, the researchers evaluated the relevance of the titles and abstracts. Each paper that met the requirements for inclusion underwent a careful examination by the reviewers. The authors talked about methods for resolving disputes. The approved study was uploaded using a data extraction form already created. The authors extracted data about the study titles, authors, study year, country, sample size, mean age, gender, and population type. A separate sheet was created for the risk of bias assessment.

Selection Criteria

Studies that reported dichotomous outcomes of vitamin D insufficiency in elderly subjects were restricted to case-control, cohort, and cross-sectional studies with a control group. The Endocrine Society guidelines categorized vitamin D insufficiency as circulating 25(OH)D ≤ 20 ng/mL (≤50 nmol/L) [15]. Studies based on the parameters of the different inflammatory events, such as disease activity, severity, duration, or region/extent of involvement, were not excluded from our analysis.

Risk of Bias

The Newcastle-Ottawa Scale (NOS) [16] for non-randomized studies (case-control and cohort studies) was utilized to evaluate the quality of the included papers. The following things were evaluated: (1) sufficient case definition: inflammatory bowel disease cases require confirmation by radiographic, histological, and clinical means; (2) the defined cases' representativeness; (3) selection criteria applied to controls; (4) cases and controls are comparable, and the study determined that age and sex were the most significant matching factors; (5) the procedure for determining exposure involves measuring the levels of vitamin D in both cases and controls.

Statistical Analysis

Comprehensive Meta-Analysis (version 3.0, Biostat, Inc., Englewood, NJ) was the software used for data analyses [17]. Using 95% confidence intervals (CIs), standardized mean differences were computed while accounting for small sample sizes (Hedges' g) [18]. Hedges' g was estimated to be big at 0.8, medium at 0.5, and small at 0.2 [19]. The cutoff point for statistical significance was P < 0.05. Prior to determining the pooled effect size and evaluating the relative contributions of all the studies in the meta-analysis, a sensitivity analysis was carried out, removing each study one at a time. Cochran's Q and I2 statistics were used to examine effect size heterogeneity. While the I2 statistics measured the percentage of variance in observed effects that reflected variance in genuine effects rather than sampling error [20,21], a statistically significant Q value (p < 0.05) revealed heterogeneity across studies [18,22].

Results

Search Results

**Figure 1:**
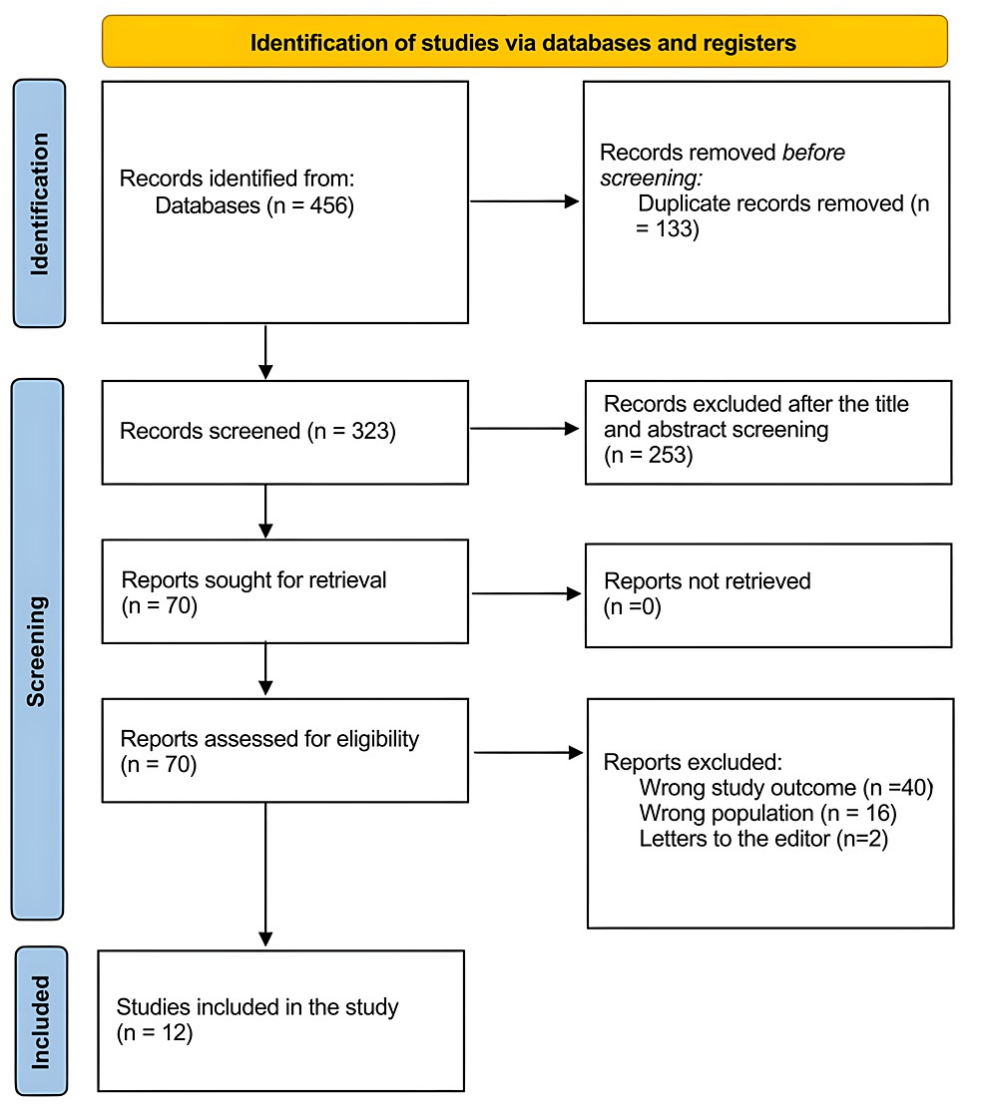
PRISMA flowchart summarizing the study selection process. PRISMA: Preferred Reporting Items for Systematic Reviews and Meta-Analyses.

A total of 456 study articles resulted from the systematic search, and 133 duplicates were deleted. Title and abstract screening were conducted on 323 studies, and 253 were excluded. A total of 70 reports were sought for retrieval, and no articles were retrieved. Finally, 70 studies were screened for full-text assessment; 40 were excluded for wrong study outcomes, 16 for the wrong population type, and two articles were letters to the editors. Twelve eligible study articles were included in this systematic review. A summary of the study selection process is presented in Figure 1.

Characteristics of the Included Studies

Table 1 presents the sociodemographic characteristics of the included study articles [23-34]. A total of 14,717 elderly patients were included in this analysis. Four studies were conducted in China [25,27,28,31], three in Italy [32-34], two in the USA [25,29], one in Germany [24], and one in Ireland [30]. This analysis included the following populations: elderly with raised inflammatory markers [30,31,34], anemia with inflammation [23,26], diabetic and metabolic syndrome subjects [24,25,28,32], chronic low back pain mediated by inflammatory markers [27], inflammation-linked vascular endothelial dysfunction [29], and ulcerated melanoma with systemic inflammation [33]. Regarding risk of bias (ROB) assessment, the NOS scores ranged from 6 to 7 in all the included studies [23-34].

**Table 1:** Sociodemographic characteristics of the included studies. NOS: Newcastle-Ottawa Scale; CVD: cardiovascular disease; NM: not mentioned.

Study ID	Study design	Country	Sample size	Mean age	Gender (M)	Population type	NOS
Indriani et al. (2018) [23]	Case-control	Indonesia	20	65.13	7 (14.5%)	Anemia with inflammation	7
Šebeková et al. (2015) [24]	Cross-sectional	Germany	266	65	148 (55.6%)	Markers of inflammation in diabetic subjects	7
Lu et al. (2022) [25]	Cross-sectional	China	200	NM	NM	Idiopathic membranous nephropathy with high inflammatory markers	7
Perlstein et al. (2011) [26]	Cohort	USA	99	70.2	NM	Anemia with inflammation	6
Hao-Wei et al. (2021) [27]	Retrospective	China	138	63.42	55 (39.9%)	Chronic low back pain mediated by inflammatory markers	7
Ma et al. (2020) [28]	Case-control	China	10389	76.4 ± 13.3	NM	Diabetic patients with the pro-inflammatory effect of homocysteine	7
Jablonski et al. (2011) [29]	Cohort	USA	75	50-79 (range)	47 (62.7%)	Inflammation-linked vascular endothelial dysfunction	7
Laird et al. (2014) [30]	Observational study	Ireland	957	70.5	481 (50.3%)	Elderly with raised inflammatory markers	7
Cheng et al. (2022) [31]	Case-control	China	180	73.33 ± 5.55	90 (50.0%)	Elderly with mild cognitive impairment raised inflammatory markers	6
Verdoia et al. (2021) [32]	Cohort	Italy	1472	69.5	975 (66.2%)	Patients with CVD, metabolic syndrome, and elevation of cellular, and humoural inflammatory parameters	6
Dozio et al. (2015) [33]	Cohort	Italy	54	66	54 (100%)	Ulcerated melanoma and systemic inflammation	7
De Vita et al. (2014) [34]	Cross-sectional	Italy	867	75.1 ± 7.1	377 (43.5%)	Elderly with raised inflammatory markers (IL-6)	7

Meta-Analysis of Primary Effect Size

**Figure 2:**
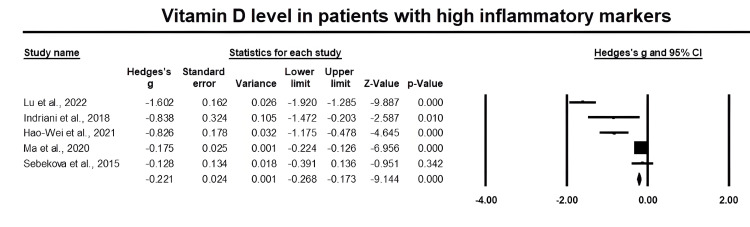
Forest plot of vitamin D levels in elderly patients with inflammatory events. References [23-25,27,28].

Figure 2 presents a forest plot of effect sizes for vitamin D levels in elderly patients with inflammatory events. There was an overall significant decrease in vitamin D levels in elderly patients with high inflammatory markers compared to controls (Hedges' g = -0.221, 95% CI: -0.268, -0.173, P < 0.001). We demonstrated significant inter-heterogeneity between studies (I2 = 95.6%, P < 0.001).

**Figure 3:**
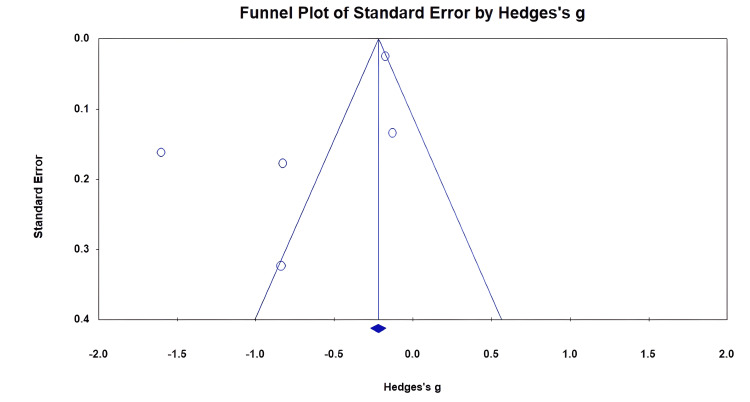
Funnel plot of publication bias detection.

Visual inspection of the funnel plot shows the asymmetrical distribution of vitamin D levels in cases and controls (Figure 3).

**Figure 4:**
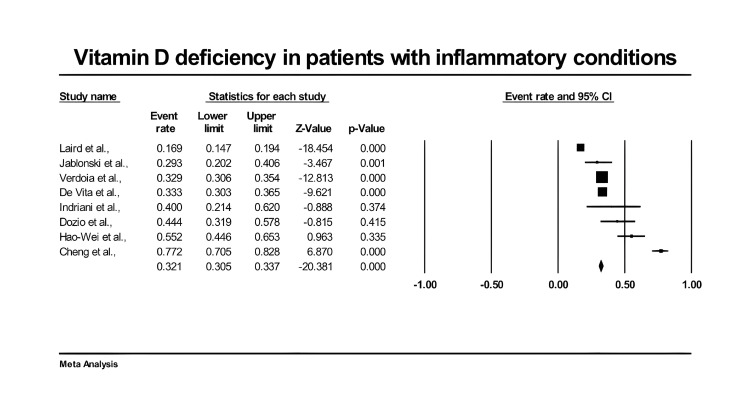
Forest plot of vitamin D deficiency in elderly patients with inflammatory events. References [23,27,29-34].

Figure 4 presents a forest plot of effect sizes for the rate of vitamin D deficiency in the elderly population with inflammatory events. There was an overall prevalence of vitamin D deficiency in elderly patients with high inflammatory markers (0.321, 95% CI: 0.305, 0.337, P < 0.001). We demonstrated significant inter-heterogeneity between studies (I2 = 97.125%, P < 0.001).

Discussion

To our knowledge, this is the first systematic review to evaluate the association between vitamin D levels and inflammatory events in the elderly population. The meta-analysis of 12 studies with data from a total of 14,717 participants showed that there was an overall significant decrease in vitamin D levels in elderly patients with high inflammatory markers compared to controls (Hedges' g = -0.221, 95% CI: -0.268, -0.173, P < 0.001). Experimental and epidemiological investigations confirm the biological plausibility of these findings. Holick et al. demonstrated that the vitamin D-supplemented groups had reduced levels of CRP, tumor necrosis factor-alpha (TNF-α), and erythrocyte sedimentation rate (ESR) in comparison to the control groups. The existence of the nuclear vitamin D receptor in almost all immune cells, such as monocytes, macrophages, and activated T and B lymphocytes, suggests that vitamin D has a role in the activity of these cytokines [35].

Additionally, T cells and monocytes from both healthy individuals and type 2 diabetes patients have their proliferation and stimulatory abilities suppressed by vitamin D, which results in the downregulation of proinflammatory cytokines like CRP, TNF-α, IL-1, IL-6, and IL-8 and the upregulation of anti-inflammatory cytokines like IL-10 [36]. Furthermore, it has been demonstrated that nuclear factor κB, a transcription factor important in inflammation and immunoregulation, functions better when the vitamin D receptor is absent, whereas vitamin D treatment inhibits nuclear factor κB translocation and reduces nuclear factor κB activity [37]. Studies on cell cultures also imply that vitamin D may have anti-inflammatory properties by focusing on signaling pathways and the cellular stress response [38].

On the other hand, in another meta-analysis of mixed demographic groups [39], vitamin D had no effect on adipokines, including leptin and adiponectin, and on CRP, TNF-α, or IL-6 in adults who were overweight or obese [40]. Additionally, vitamin D supplementation was found to have no effect on inflammation in healthy adults based on systematic reviews of randomized controlled trials without the use of meta-analyses [41,42]. The incorporation of healthy populations in prior reviews may have contributed to discrepancies between existing meta-analyses and the current review's findings. This is because it has been suggested that vitamin D has stronger effects when the immune system is boosted, such as when there is a diagnosis of inflammatory or chronic conditions [43]. The current study extends our knowledge by presenting that low vitamin D levels were significant in elderly patients with marked inflammatory events in different chronic conditions such as type 2 diabetes, anemia, vascular endothelial dysfunction, ulcerated melanoma, and other diseases characterized by systemic inflammation.

In the present meta-analysis, the overall prevalence of vitamin D deficiency in elderly patients with high inflammatory markers (0.321, 95% CI: 0.305, 0.337, P < 0.001). We demonstrated significant inter-heterogeneity between studies (I2 = 97.125%, P < 0.001). It has been found that vitamin D deficiency is highly prevalent in the senior population. It is estimated that between 40% and 100% of senior citizens worldwide may be vitamin D deficient. American values have improved somewhat as a result of food fortification. Elderly people who are institutionalized or homebound have greater rates of vitamin D deficiency [44,45]. This connection is especially significant for the older population, whose skin production of vitamin D produced by ultraviolet light may be less than ideal. Age-related alterations in non-adaptive immunity, such as thinning skin, enlarged prostate, reduced cough reflex, and other anatomic or physiological accompaniments, make older people more susceptible to infection [46].

Our study has several limitations. Firstly, the entire set of data was observational, and observational data are prone to bias due to unmeasured confounders. Secondly, there is high inter-heterogeneity between the included studies, which may be due to the inclusion of various diseases with high inflammatory markers. We did not conduct subgroup analysis for these conditions due to the limited number of the included studies. Thirdly, we did not include subgroup analysis for each inflammatory marker in association with vitamin D deficiency.

## Conclusions

This systematic review and meta-analysis demonstrated a significant decrease in vitamin D levels among the elderly with different inflammatory conditions. Additionally, the rate of vitamin D deficiency was comparable to the global rates of vitamin D deficiency among the elderly population. Future longitudinal studies and well-designed, large, randomized controlled trials are required to study the association between vitamin D deficiency and the incidence of inflammatory events in this specific group of patients.
